# School Coordinators’ Perceptions of Organizational Readiness Is Associated with Implementation Fidelity in a Smoking Prevention Program: Findings from the X:IT II Study

**DOI:** 10.1007/s11121-020-01197-1

**Published:** 2021-01-06

**Authors:** Lotus Sofie Bast, Henriette Bondo Andersen, Anette Andersen, Stine Glenstrup Lauemøller, Camilla Thørring Bonnesen, Rikke Fredenslund Krølner

**Affiliations:** 1 University of Southern Denmark, Odense, Denmark; 2 Steno Diabetes Center, Gentofte, Denmark

**Keywords:** School organizational readiness, Implementation, Smoking prevention, School intervention, Motivation, Capacity

## Abstract

School organizational readiness to implement interventions may play an important role for the actual obtained implementation level, and knowledge about organizational readiness prior to intervention start can help pinpoint how to optimize support to the schools. In this study, we applied a novel heuristic, *R* = MC^2^ to assess school organizational readiness prior to implementation of a multicomponent smoking prevention program. Furthermore, we examined the association to actual implementation after the first year of study. We used questionnaire data from school coordinators at 40 schools in Denmark who had accepted to implement the multi-component smoking prevention intervention—X:IT II—in the school year 2017–2018 including three main components: (1) Rules on smoke-free school time, (2) A smoke-free curriculum, and (3) Parental involvement. On behalf of the school, a school coordinator answered a baseline questionnaire about the organizational readiness and a follow-up questionnaire about implementation of the three components after first year of study. Readiness was measured by summing aspects of motivation (relative advantage, compatibility, complexity, and priority), general capacity (culture, climate, and staff capacity), and innovation-specific capacity (knowledge, skills, and abilities). Based on school coordinators’ perceptions, almost all schools had good general capacity while the other two areas of readiness varied across schools; overall, 56.8% of schools (*N* = 25) had good motivation for implementing the X:IT II intervention and 61.3% (*N* = 27) had high innovation-specific capacity. Half of the schools had high overall readiness defined as high motivation and high innovation-specific capacity. Schools with high overall readiness implemented the rules on smoke-free school time, smoke-free curriculum, and parental involvement to a higher degree than schools with low overall readiness. All participating schools possessed sufficient levels of general capacity, e.g., a well-functioning organizational culture and sufficient staff capacity. High levels of motivation and innovation-specific capacity were positively associated with the schools’ actual implementation of the main intervention components. This way of conceptualizing and measuring organizational readiness may be useful in future studies, i.e., in studies where enhancing readiness is a main objective.

## Introduction

School-based smoking prevention is an important and widely used strategy for reducing the overall smoking prevalence in youth (Thomas et al. 2013). The past years, a long-lasting decline in smoking rates among Danish youth has been replaced by stagnation (Rasmussen et al. 2018). New data even imply an increase among some groups (Hoffmann et al. 2018). Together with structural initiatives for preventing smoking, school-based initiatives have the potential to change this worrying trend (Vestbo et al. 2018). Despite this potential, school-based smoking prevention—as well as other school-based interventions—often suffers from poor implementation and variation in effect (Domitrovich et al. 2008). It is well documented that implementation levels influence outcomes of interventions (Fixsen et al. 2009; Naylor et al. 2015; Durlak and DuPre 2008), also within smoking prevention (Bast et al. 2016). School-based interventions are often multi-component and designed to function on several levels, i.e., the school, the school class, and the individual level (Domitrovich et al. 2008). Schools’ level of implementation of these multi-component interventions may vary, i.e., some schools may implement all components well, while others may only be successful in implementing one specific component (Bast et al. 2016). This suggests that schools have different needs for support depending on the nature of the intervention component.

Implementation levels are influenced by characteristics of the intervention (i.e., complexity of the intervention) and the surrounding intervention support system (Domitrovich et al. 2008). The intervention support system serves to establish the necessary conditions for delivery of the intervention, i.e., pre-intervention training given to teachers, or coaching and mentoring of teachers during the implementation process. Therefore, the intervention and the support system are independent, although interrelated, components of a whole (Domitrovich et al. 2008).

Furthermore, schools’ organizational readiness to implement interventions may also play an important role for the actual obtained level of implementation, and knowledge about readiness prior to intervention start can pinpoint how to optimize support to the schools (Scaccia et al. 2015). Organizational readiness to implement refers to the extent to which an organization is both willing and able to implement a new initiative (Scaccia et al. 2015). There is no consensus about the conceptualization and measurement of organizational readiness (Miake-Lye et al. 2020, Livet et al. 2019, Weiner et al. 2020). Traditionally, most scholars have viewed readiness as a psychological construct; however, recently, Scaccia et al. (2015) have proposed that readiness consists of both psychological and structural dimensions (Weiner et al. 2020). Hence, Scaccia and co-authors (2015) suggest, that the overall readiness is a function of the organizations’ (1) motivation to implement a specific intervention, (2) general capacity, and (3) innovation-specific capacity (Scaccia et al. 2015). They argues that if one of these areas is close to zero, high degrees on the two other areas will not make an organization ready to implement the intervention and proposes a *R* = MC^2^ heuristic (readiness = motivation * general capacity * innovation-specific capacity) (Scaccia et al. 2015). Traditionally, organizational readiness was related to the initial phases of implementation (Meyers et al. 2012; Wanless and Domitrovich 2015); however, in the *R* = MC^2^, framework readiness is viewed as a matter throughout the life cycle of implementation, hence a departure from a binary “ready or not” approach (Domlyn and Wandersman 2019). Furthermore, organizations may be high in some areas of readiness, i.e., motivation, but at the same time low in others. The different areas may interact and thereby strengthen or weaken each other. Still, knowledge about the relative importance of the three areas is sparse (Scaccia et al. 2015).

**Organizational motivation** is related to the specific intervention being implemented. It includes beliefs and expectations related to the intervention, such as anticipated outcomes of participation, the complexity of the intervention, and the compatibility between the intervention and the organizational values and norms (Scaccia et al. 2015).

**General organizational capacity** relates to the overall functioning of an organization and is associated with the ability to implement *any* intervention. It is not related to the specific intervention being implemented but comprises aspects of how things are routinely done in the organization. Important aspects of general capacity include the organizational culture and climate and the staff capacity (Scaccia et al. 2015).

**The innovation-specific capacity** is directly related to conditions that are important for implementing the specific intervention. Therefore, the important aspects may vary depending on the nature of the specific intervention; some interventions are simple, while others are more complex. Important aspects are the knowledge, skills, and abilities required and whether there are staff members willing and able to facilitate the implementation (Scaccia et al. 2015).

Most previous implementation research has focused on the innovation-specific capacity rather than organizational motivation and general capacity (Scaccia et al. 2015). This means that important barriers may be overlooked. If for example a school has good innovation-specific capacity, but the overall functioning of the school is bad, the chance that a new intervention will be adopted and implemented is doubtful.

Research on school readiness is still in its infancy and few school-based health intervention studies have applied Scaccia’s heuristic (Kingston et al. 2018). Successful adoption, implementation, and up-scaling of interventions require that we understand both why and how schools manage to implement preventive initiatives, as well as their capacity to implement (Allen et al. 2017). By assessing schools’ organizational readiness prior to implementation, it may be possible to understand and support schools in the areas most needed. Furthermore, studies on the association between readiness and actual implementation are needed (Allen et al. 2017; McKay et al. 2019).

The aim of this study was to apply Scaccia’s heuristic (*R* = MC^2^) to (1) assess organizational readiness at schools accepting to participate in the smoking preventive X:IT II study prior to implementation of the multicomponent intervention, and (2) examine the association between school organizational readiness and actual implementation of the three main intervention components after first year of study.

This study has been pre-specified in Current Controlled Trials ISRCTN31292019.

## Methods

### The X:IT II Study

We used data from the X:IT II study, which evaluates the effect of the X:IT II intervention in 46 schools in Denmark in 2017–2020 (Bast et al. 2019).

The X:IT intervention was originally launched by the Danish Cancer Society in 2010 (Andersen et al. 2014). The evaluation of this first version showed a significant lower risk of smoking among students at intervention schools compared to control schools (odds ratio = 0.61, CI: 0.45–0.82) (Andersen et al. 2015). However, a qualitative process evaluation suggested that certain aspects of the intervention components did not appeal to participants from lower socioeconomic classes. This was mainly due to the wording being too academic and that smoking parents could not recognize themselves or their children in the materials. Based on this information, the intervention components were modified and tested in a new sample of schools from 2017 to 2020 (Bast et al. 2019).

### The X:IT II Intervention

The X:IT II intervention consists of three main intervention components:

- *Smoke-free school time:* The rules for smoking at X:IT II intervention schools are stricter than the regular rules for schools directed by the national law; intervention schools are encouraged to secure that there is no smoking anywhere, neither on the school ground nor outside the school ground during school hours. This applies to all students, teachers, other employees, and visitors in the schools (Bast et al. 2019). In Denmark, some schools outside the evaluation of X:IT II has applied smoke-free school time voluntarily, while most schools have not. Implementing smoke-free school time requires support from the school leader as well as the other employees. Especially in the beginning, there need to be put a lot of resources into enforcing the rules.
- *Smoke-free curriculum:* The educational material “Up in Smoke” (webpage: www.opiroeg.dk) was developed to target students in grades 7 to 9. It is cross-curricular and includes eight lessons a year for three years. The material was developed to fit into the ordinary school activities, in order not to impose extra workload on teachers.
- *Parental involvement:* Parents are encouraged to sign smoke-free agreements with their children. And use the opportunity to talk to their children about tobacco. For this purpose, a website was developed (www.snakomtobak.dk, in English; Chat on Tobacco). The website has different entrances that target various groups of parents: smoking and non-smoking parents, and parents with and without children that smoke. Teachers should introduce the webpage and the X:IT II project in general to parents at parent-teacher meetings in the beginning of the school year.

For a detailed description about the intervention components, see Bast et al. (2019).

### Study Design

Development and implementation of the X:IT II study were carried out by the Danish Cancer Society and process and effect evaluation was conducted by a research group at the Centre for Intervention Research, University of Southern Denmark.

Sampling and recruitment of schools took place during spring 2017 in collaboration between the Cancer Society and Centre for Intervention Research. Initially, 57 schools agreed to participate in the evaluation. After the summer holiday, just before the data collection was about to start, 11 of the schools withdrew from the evaluation; two schools had hired new school leaders who did not want to participate; two schools had committed themselves to too many projects; seven schools mentioned lack of time as the primary reason for resigning. Therefore, baseline data included 46 schools from all over Denmark (Bast et al. 2019).

Each participating school assigned a school coordinator, most often a 7th grade teacher to be responsible for coordinating intervention activities at school and informing colleagues about the intervention. Furthermore, the school coordinator handled the data collections among students and responded to a school coordinator questionnaire four times during the evaluation period.

In this study, we used school coordinator responses to a baseline questionnaire on organizational readiness collected before the intervention start (August 2017), and a follow-up questionnaire on implementation of main components at the end of the first study year (May to June 2018) (Bast et al. 2019). The school coordinators were chosen as respondents as they were considered a key informant on the schools’ decision to adopt and implement the intervention.

**Fig. 1:**
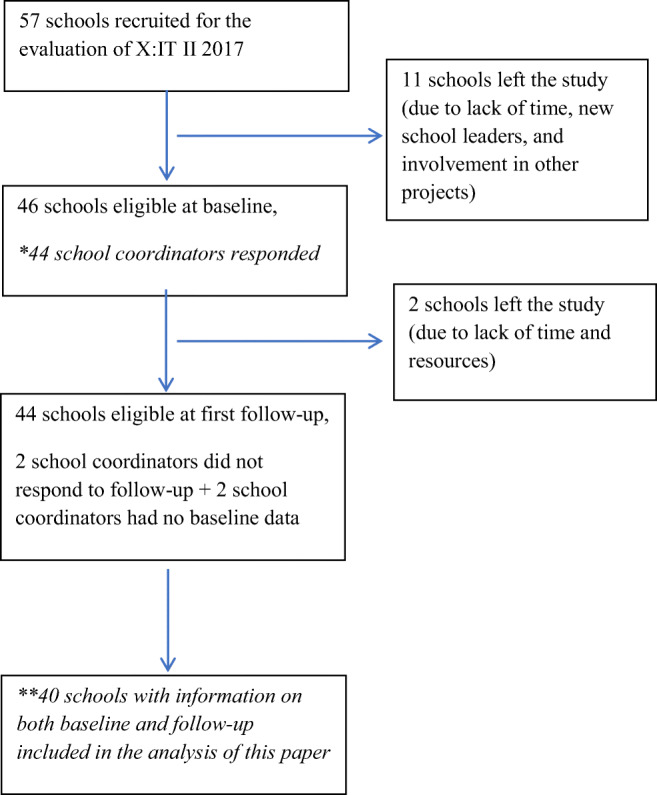
Flow diagram of schools included in the evaluation of the 2017 X:IT II intervention. Single asterisk means included in the assessment of readiness, and double asterisks mean included in the implementation assessment

Of the 46 schools invited to the baseline survey, two school coordinators did not respond to the questionnaire and were therefore excluded from this study. The analysis of organizational readiness included 40 schools with both baseline and follow-up measures (see Fig. 1).

### The Setting

In Denmark, children start primary school the year they turn six. There is one year of preschool and nine years of regular schooling. There is no grouping by ability, and 85% of all children attend the public schools. Each school class has several different teachers as most teacher teach a limited number of subjects. Generally, the older the students are, the more teachers are involved in teaching the class.

Compared to most countries in the Western world, Denmark has a very lenient smoking policy (Jarlstrup et al. 2018). Cigarette prices are still relatively low, and the first law restricting smoking in public places was adopted in 2007. By August 2012, smoking was fully banned all over the school grounds for students, employees, and visitors at schools with students under the age of 18.

### Measures

#### Measures of Organizational Readiness for Implementation

The development of the baseline questions on school organizational readiness was based on the framework by Scaccia et al. (2015). Measures of organizational readiness applied in the X:IT II study, response categories, and criteria for satisfying level are summarized in Table 1.

**Table 1:** Definitions and measurements of factors that influence motivation, general capacity, and innovation-specific capacity for the X:IT intervention

Component	Definition	Measured in X:IT	Response categories	Criteria for satisfying level
Motivation				
Relative advantage	Degree to which the innovation is perceived as being better than what it is being compared against	“It seems that there are clear advantages using X:IT compared to other school-based smoking preventive programs”	Totally agree; agree; neither; disagree; totally disagree	Totally agree + agree
Compatibility	Degree to which the innovation is perceived as being consistent with existing values and norms	“X:IT is compatible with the existing school values on smoking”	Totally agree; agree; neither; disagree; totally disagree	Totally agree + agree
Complexity	Degree to which the innovation is perceived as relatively difficult to understand and use	1. “It seems that the teaching materials are easy to work with” 2. “It seems that fulfilling the demands for smoke-free school time for teachers will be difficult” 3. “It seems that fulfilling the demands for smoke-free school time for students will be difficult”	Items 1–3: Totally agree; agree; neither; disagree; totally disagree	Item 1: Totally agree + agree Items 2–3: Disagree + totally disagree
Priority	Extent to which the innovation is regarded as more important than others	“Among other things, it is a school responsibility to work with smoking prevention”	Totally agree; agree; neither; disagree; totally disagree; we have not talked about this	Totally agree + agree
General organizational capacity				
Culture	Expectations about how things are done in an organization	“Teachers in my school collaborate well” “Conflicts between teachers are handled well”	Totally agree; agree; neither; disagree; totally disagree;	Totally agree + agree
Climate	Collective feeling about current working environment	“School employees generally like each other” “School employees can express their feelings and opinions”	Totally agree; agree; neither; disagree; totally disagree	Totally agree + agree
Staff capacity	General skills, education, and expertise that the staff possesses	During the last years, has your school: “Talked about smoking prevention at meetings?” “Purchased teaching resources about smoking prevention?”	Yes; no; do not know	Yes
Innovation-specific capacity				
Innovation-specific knowledge, skills, and abilities	Knowledge, skills, and abilities needed for the innovation	“I feel prepared to handle the X:IT school coordinator role”	Totally agree; agree; neither; disagree; totally disagree	Totally agree + agree
Program champion	Individual(s) who put charismatic support behind an innovation through connections, expertise, and social influence	“I was involved in the decision of being the X:IT school coordinator”	Totally agree; agree; neither; disagree; totally disagree	Totally agree + agree
Specific implementation climate supports	Extent to which the innovation is supported; presence of strong convincing, informed, and demonstrable management support	1. “My school leader supports the X:IT project, inclusive the anti-smoking rules at school” 2. “My school leader supports me in the function as X:IT school coordinator”	1–2: Totally agree; agree; neither; disagree; totally disagree	1–2: Totally agree + agree

**Organizational motivation** was measured by the following four aspects as suggested by Scaccia et al. *relative advantage*, *compatibility*, *complexity*, and *priority* (Table 1).

Based on conceptual discussions prior to analyses, we categorized schools as having high motivation for implementing the X:IT II intervention if they agreed on at least half of the items on motivation—that is at least four out of six items. The readiness heuristic does not provide guidance about this conceptualization; instead, we were inspired by the implementation fidelity literature, where a rule of thumb is that an implementation level of around 60% is realistic and acceptable.

**General capacity** was measured by aspects of *culture*, *climate*, and *staff capacity* (Scaccia et al. 2015). High general capacity was defined as agreeing on at least half of the items (at least four out of six).

**Innovation-specific capacity** was measured by aspects of innovation-specific *knowledge*, *skills*, and *abilities*, the presence of *a program champion*, and *specific implementation climate support* (Scaccia et al. 2015). High innovation-specific capacity was defined as agreeing on at least three of the four items about innovation-specific capacity.

**Overall readiness:** Due to lack of variation between schools on general capacity, we decided to only include innovation-specific capacity and motivation in the analyses of association to implementation.

#### Measures of Implementation of Main Components

**Smoke-free school time** was measured by items on smoking rules at school for students: “Are students allowed to smoke during school time?” with responses; “Yes, students can smoke in a special area,” “Yes, students can smoke outside the school ground,” and “No, students are not allowed to smoke no matter where they are” and employees: “Are employees allowed to smoke during school time?” with responses “Yes, in a special area, visible to students,” “Yes, in a special area, not visible for students,” “Yes, outside school grounds, visible for students,” “Yes, outside school grounds, not visible for students,” and “No, no one can smoke no matter where they are.”

Schools were only categorized as fulfilling the rules for smoking if they had rules for smoke-free school time for both students and employees, i.e., responded no smoking allowed for students and employees no matter where they are.

For **curricular activities**, coordinators were asked whether all 7th grade classes had received the mandatory eight lessons about smoking and tobacco during the first year. Schools that responded “Yes, all” and “Yes, some of the classes” was categorized as having implemented the component, whereas “No” + “Less than eight lessons” + “Do not know” were categorized as not implemented.

**Parental involvement** was measured by two items; one about presentations at parental meetings and one about smoke-free agreements. School coordinators responded to the item: “Was X:IT presented at parent teacher meetings in 7th grade classes?”, here “Yes, in all classes” + “Yes, in some of the classes” was categorized as implemented, whereas “No” and “Do not know” was categorized as not having implemented. The item on smoke-free agreements was as follows: “Students in 7th grade is surveyed as part of the evaluation of X:IT II. Did students in 8th and 9th grade also receive the smoke-free agreements?” Here implementation was “Yes, all” + “Yes, some of the classes”, whereas “No” + “Do not know” was not implemented.

### Data Analysis

School was the unit of analysis. The definition of satisfying levels for the areas of readiness (organizational motivation and innovation-specific capacity), as well as implementation of the three main components (smoke-free school time, curricular activities, and parental involvement) was based on conceptual discussions, and cut points were decided a priori to the analyses. Sensitivity analyses using different cut points for dichotomizations showed results in the same direction.

Results are presented in a radar plot; a graphical method well suited for presenting differences or similarities between groups in multiple variables (Saary 2008). The plot is shaped as a circle: from the center comes a number of rays, each representing a variable. Here, the rays represent implementation of the main intervention components, i.e., smoke-free school time. Each group of schools (high vs. low readiness) has its own marking in the plot. Details about included variables and dichotomizations are shown in Table 1.

## Results

School readiness to implement the X:IT II intervention varied which is presented below. Results for each school are shown in Table 2.

**Table 2:** Motivation, general organizational, innovation-specific capacity, and overall school readiness for each school in the X:IT II study

	Motivation					General organizational capacity				Innovation-specific capacity				
School	Relative advantage	Compatibility	Complexity (teaching/rules teachers/rules students)	Priority	Overall motivation, *H* ≥ 4	Culture	Climate (like/conflicts)	Staff capacity (meetings/teaching)	Overall general capacity, *H* ≥ 4	Knowledge, skills, abilities	Program champion	Specific implementation climate supports (project/coordinator)	Overall innovation-specific capacity, *H* ≥ 3	School readiness**
1	+	–	±/−	–	L	±	±	−/−	L	+	+	−/−	L	LL
2	+	+	+/+/−	+	H	+/+	+/+	−/−	H	+	+	+/+	H	HH
3	+	+	+/−/+	+	H	+/+	+/+	−/−	H	–	–	±	L	HL
4	+	+	+/+/+	+	H	+/+	+/+	−/−	H	+	+	+/+	H	HH
5	–	+	+/+/+	+	H	+/+	+/+	+/+	H	–	+	+/+	H	HH
6	–	+	−/−/−	+	L	+/+	+/+	−/−	H	–	–	+/+	L	LL
7	+	+	+/+/+	+	H	+/+	+/+	−/+	H	–	+	+/+	H	HH
8	+	+	+/+/+	+	H	+/+	+/+	−/+	H	+	–	+/+	H	HH
9	+	–	+/−/+	–	L	+/+	+/+	−/+	H	–	–	−/−	L	LL
10	–	+	−/−/−	+	L	+/+	+/+	−/+	H	–	+	./.	L	LL
11*	–	+	+/−/−	+	L	+/+	+/+	−/−	H	–	–	+/+	L	LL
12	+	+	+/+/+	+	H	+/+	+/+	+/−	H	+	+	+/+	H	HH
13	+	+	+/+/+	+	H	+/+	+/+	−/+	H	+	+	+/+	H	HH
14	+	+	+/+/+	+	H	+/+	+/+	+/+	H	–	–	./.	L	HL
15	–	–	−/−/−	–	L	+/+	+/+	+/−	H	–	+	+/+	H	LH
16	+	+	+/+/+	+	H	+/+	+/+	−/−	L	+	+	+/+	H	HH
17	+	+	+/−/−	+	H	+/+	+/+	−/+	H	+	+	+/+	H	HH
18	–	+	+/−/+	+	H	±	+/+	±	H	+	+	+/+	H	HH
19	–	–	−/−/+	–	L	+/+	+/+	−/−	H	–	–	−/+	L	LL
20	–	+	−/−/−	+	L	+/+	+/+	−/−	H	+	+	+/+	H	LH
21	–	+	+/+/+	+	H	+/+	+/−	−/−	L	–	–	−/−	L	HL
22	+	+	+/+/−	+	H	+/+	+/+	+/+	H	+	+	+/+	H	HH
23	–	–	−/−/+	+	L	+/+	+/+	−/−	H	–	–	+/+	L	LL
24	–	+	−/−/−	+	L	+/−	−/+	−/+	L	–	–	+/+	L	LL
25	–	+	−/−/+	+	L	+/+	+/+	−/−	H	+	+	+/+	H	LH
26	+	+	+/−/+	+	H	+/+	+/+	+/+	H	+	+	+/+	H	HH
27	–	+	+/+/+	+	H	+/+	+/+	−/−	H	+	+	+/+	H	HH
28	+	+	−/+/+	+	H	+/+	+/+	+/+	H	+	+	+/+	H	HH
29	+	+	+/−/+	+	H	+/+	+/+	+/−	H	–	+	+/+	H	HH
30	+	+	+/+/+	+	H	+/−	+/+	+/−	H	+	–	+/+	H	HH
31	+	+	+/−/−	+	H	+/+	+/+	−/−	H	–	–	+/+	L	HL
32	+	+	+/−/−	+	H	+/+	+/+	−/−	H	+	+	+/+	H	HH
33	+	+	+/+/+	+	H	+/+	+/+	−/−	H	+	+	+/+	H	HH
34	+	+	+/+/+	+	H	+/+	+/+	+/+	H	+	+	+/+	H	HH
35	–	+	−/−/−	+	L	+/+	+/+	+/+	H	–	+	−/−	L	LL
36	+	–	−/−/−	+	L	−/+	+/+	−/−	L	+	+	+/+	H	LH
37	+	+	+/+/+	+	H	+/+	+/+	+/+	H	+	+	+/+	H	HH
38	+	–	+/−/−	+	L	+/−	+/+	+/−	H	–	+	+/−	L	LL
39	–	+	−/+/+	+	H	+/+	+/+	+/−	H	–	+	+/+	H	HH
40*	–	–	−/−/−	+	L	−/+	+/+	−/−	L	–	+	+/+	H	LH
41*	–	–	−/−/−	–	L	+/+	+/+	−/−	H	–	–	−/−	L	LL
42*	–	+	−/−/−	+	L	±	+/+	+/.	H	–	–	+/+	L	LL
43	–	+	−/+/−	+	L	+/+	+/+	−/−	H	–	+	+/+	H	LH
44	–	–	−/−/+	+	L	+/+	+/+	−/−	H	+	+	+/+	H	LH
*N* schools (%)	24 (54.5)	34 (77.3)	27 (61.3)/19 (43.2)/25 (56.8)	39 (88.6)	25 (56.8)	42(95.5)/38 (86.4)	43 (97.7)/42 (95.5)	16 (36.4)/15 (34.1)	39 (88.6)	22 (50.0)	30 (68.2)	36 (81.8)/35 (79.5)	27 (61.3)	HH:21 (47.7) LL:12 (27.3) HL/LH:11 (25.0)

“+” indicates satisfying level for organizational readiness

*Schools participating at baseline but dropped out before first follow-up or did not respond to the follow-up questionnaire

**School readiness based on organizational motivation and innovation-specific capacity: *HH*, high; *LL*, low; *HL/LH*, medium

### Organizational Motivation

Overall, 56.8% (25 schools) had good motivation for implementing the X:IT II intervention (Table 2). More than half of the school coordinators agreed that there were relative advantages to implementing the X:IT II intervention compared to other school-based smoking preventive initiatives. Most school coordinators agreed that X:IT II was compatible with existing school values (77.3%) and that it was a school responsibility to work with smoking prevention (88.6%). Regarding perceived complexity of the intervention, 61.3% of school coordinators responded that the educational materials seemed easy to work with, whereas half of the school coordinators were concerned about whether they would be able to adhere to the rules for smoke-free school time.

### General Organizational Capacity

Most participating schools had good general capacity; 88.6% (39 schools). Most schools responded positively on the items about culture and climate, i.e., teachers were good at collaborating with each other and teachers generally liked each other. Few schools had discussed smoking at meetings or purchased teaching resources about smoking prevention during the last years.

### Innovation-Specific Capacity

High innovation-specific capacity was found in 61.3% of schools (27 schools). Half of the school coordinators responded that they felt prepared to handle the role as X:IT coordinator, and two-thirds that they were involved in the decision of being the school coordinator. Most school coordinators felt supported by the school leader.

### Overall Readiness

Almost half of the schools (47.7%) were high on readiness (motivation and innovation-specific capacity), whereas one-fourth (27.3%) had low readiness (Table 2).

### Implementation

Implementation data are provided in Table 3. Regarding rules for smoking at school, 72.5% of schools (29 schools) had smoke-free school time for students, and 40.0% (16 schools) had smoke-free school time for employees. Overall, 35.0% (*N* = 14) had smoke-free school time for all. In 67.5% of schools (*N* = 27), the curricular activities about smoking and tobacco were provided to some or all school classes, in 32.5% of schools (*N* = 13) classes did not receive the required lessons. Overall, the smoke-free agreements were implemented among students at 35.0% of the schools (*N* = 14) and 67.5% of schools (*N* = 27) presented X:IT at parent-teacher meetings.

**Table 3:** Implementation of main intervention components after first year of the X:IT II study

	Implemented, number of schools	Not implemented (missing)
Smoke-free school time		
For students	29	11
For employees	16	23 (1)
Smoke-free school time for all	14	
Teaching		
Mandatory eight lessons	27	13
Parental involvement		
Smoke-free agreements	14	24 (2)
Parent-teacher meetings	27	12 (1)

### Overall Readiness and Implementation

**Fig. 2:**
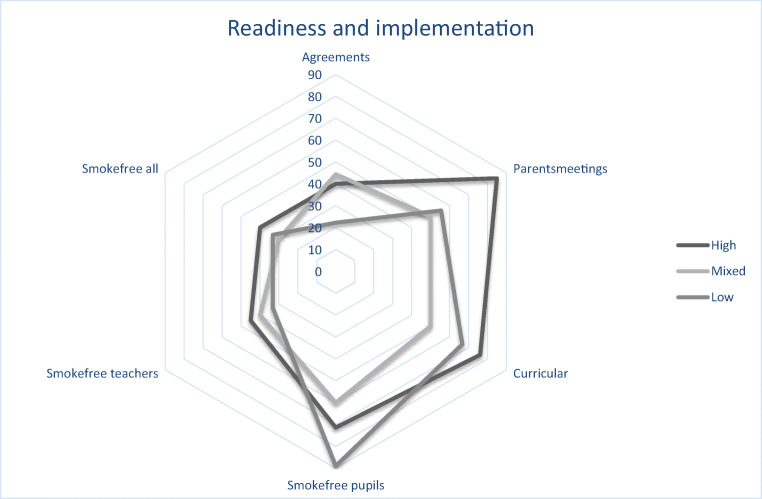
Association between readiness and implementation of the main intervention components in the X:IT II study at first year follow-up

Schools with high motivation and high innovation-specific capacity (= high readiness) seemed to implement the educational materials, parental meetings, smoke-free agreements, and smoking rules for teachers to a higher degree than schools which were low on both areas (Fig. 2). Schools with low readiness had firmer rules for student smoking at school.

## Discussion

Our study is one of the first studies to apply the novel measure of organizational readiness to implement suggested by Scaccia et al. (2015) in a school-based intervention study and examine its association with implementation level. Hence, we examined three areas of organizational readiness to implement the X:IT II intervention and found high levels of general capacity and varying levels of motivation and innovation-specific capacity among participating schools. As previously stated, Scaccia et al. argue that if one of the areas in the organizational readiness heuristic is close to zero, high degrees on other areas of readiness will not make an organization ready to implement the intervention (Scaccia et al. 2015).

The overall high level of general capacity may reflect that schools with low general capacity decline to participate in intervention studies. A relatively large group of schools declined to participate in the X:IT II study due to lack of time and resources—aspects that might imply low general capacity. This is supported by the Delphi study by Domlyn and Wandersman (2019) suggesting that general capacity is most important in the pre-adoption and adoption phase. General capacity was excluded from the overall readiness measure in the examination of an association to implementation due to lack of variance between schools. Using the overall readiness measure—defined as having both high motivation and high innovation-specific capacity—approximately half of participating schools turned out to have high readiness for implementing the X:IT II intervention prior to intervention start. In other words, these schools, theoretically, had an optimal starting point.

As also seen in previous studies (Durlak and DuPre 2008; Bast et al. 2016), the three intervention components were not fully implemented when assessing implementation ten months later. However, we found that schools with high readiness had implemented the educational materials, parental meetings, smoke-free agreements, and smoking rules for teachers to a higher degree than schools which were low on both motivation and innovation-specific capacity. Surprisingly, rules for student smoking at school were stricter in schools with low readiness, this may reflect that schools with low readiness have a stronger need for clear rules for student smoking, compared to schools with high readiness.

Previous school-based interventions have found that the level of implementation changed during the intervention period and complex interventions may take several years to be implemented properly (Felner et al. 2001; Meyers et al. 2012). For example, the school-based, multi-component Boost intervention targeting 13-year-olds’ fruit and vegetable consumption found that fruit delivery from external suppliers increased over time, whereas teachers’ delivery of educational activities varied over time (Aarestrup et al. 2015). Similarly, in the first evaluation of X:IT, we found that implementation of rules for smoking increased during the study period, while the implementation of educational activities decreased over time (Bast et al. 2016). Associations found at first follow-up may therefore be different later in the intervention period. Also, the importance of each readiness area may vary over time (Domlyn and Wandersman 2019).

### Methodological Issues

The strengths of the X:IT II study include the large, nationwide study with high response rates, use of multiple data sources, and previously applied questionnaire items. We measured baseline indicators of school organizational readiness to implement before the actual implementation started.

Interestingly, we found that only a little more than half of the participating schools possessed high motivation for implementing the X:IT II intervention. That means that a relatively large part of schools was not motivated, however still agreed to participate. This may reflect a discrepancy between the persons in schools that consented to participate (often a school leader), and the X:IT coordinators who delivered the intervention and responded to the questionnaire. Results from this study should be interpreted in the light of the participation of only one school coordinator in each participating school. Different staff members may perceive aspects of readiness differently (Hustus and Owens 2018) and using more respondents in each school might give a more reliable picture of the school readiness. However, getting busy teachers to respond to questionnaires is a difficult task and low teacher response rates have been found in previous Danish school-based studies, as well as a huge variation in number of teachers responding per school (Bonnesen et al. 2020; Jørgensen et al. 2015). This suggest that though it is not ideal using the main responsible coordinator, as respondent might be the only way to assess readiness. Furthermore, to stimulate a fruitful research-practice partnership, we also wanted to respect their main task of teaching.

Teachers reported how many activities they had implemented and there might be a risk of over estimations of actual delivered intervention activities as they feel committed to the research project (social desirability bias). However, this did not seem to be the case in our study as coordinators also reported low levels of implementation. Using observations to assess level of implementation level may be a way to overcome social desirability bias; however, doing observations in many schools is a very time consuming and costly job. Furthermore, correspondence between teacher self-reported data and observational data (Abry et al. 2013; Bigss et al. 2008) imply that teacher self-reports can be a reliable measure. Moreover, teachers may be inclined to perform better while being observed than they would do under normal conditions.

Measures about aspects of motivation, general, and innovation-specific capacity were based on items previously used in other studies. Other measures could have been used, or other aspects of the three areas of readiness could have been measured, thereby theoretically illustrating a different picture of readiness and implementation. Overlooked important aspects of general capacity may be explored in qualitative studies (Arthur et al. 2020). We chose to dichotomize measures of motivation, general, and innovation-specific capacity into high or low by cut points of a positive response on at least half of the applied items. This allowed for allocation of schools into groups of high and low readiness; however, this also means that we cannot see which aspects of, i.e., motivation schools were high or low on. Future research should explore this more deeply. All aspects within motivation, general, and innovation-specific capacity weighted equally which may have masked the results. At present, there exists no guidance on whether items should be weighted differently (Wanless and Domitrovich 2015).

For the purpose of this study, we measured readiness prior to intervention start. We do recognize, though, that readiness may change over time. Studies suggest that motivation may be most important in the early phases, whereas the other areas are more relevant throughout the lifespan of the intervention (Domlyn and Wandersman 2019). Four schools dropped out between baseline and follow-up; three were low on overall readiness, whereas the last one had low motivation and high innovation-specific capacity. We have no information about readiness for schools that agreed to participate in the first place and then declined before baseline. This information could have contributed to the overall learning and understandings from this study and might be a priority for future studies.

The association between readiness and implementation may be part of the explanation why we do not always succeed with school-based initiatives. More research is needed on the relative importance of motivation, innovation-specific capacity, and general capacity and effective means to improve these areas. Most research has focused on improving innovation-specific capacity to specific innovations (Maras et al. 2014). However, research studies suggest that improving the general capacity in, e.g., a school, will also impact the ability to implement specific interventions, thereby leaving schools with better overall capacity (Flaspohler et al. 2008; Maras et al. 2014; Malloy et al. 2015).

## Implications

It is our hope that others will continue working with examining readiness and implementation based on the Scaccia heuristic (Scaccia et al. 2015), thereby contributing to develop a common language and use of methods within implementation science in the future. Combining methods, i.e., quantitative and well as qualitative, may further improve the research.

As the analyses for this paper were conducted after intervention start with the aim of examining readiness and its association to actual implementation, our assessment of organizational readiness could not be used to support schools that were low on selected aspects. All schools in the X:IT II study received the same implementation guidelines and similar levels of support. Future interventions may use the readiness assessment as an active part of the implementation process, i.e., by informing schools of their level of organizational readiness and guide them on how to use this knowledge to improve implementation. Capacity building in schools can lead to higher implementation and improved outcomes (Flaspohler et al. 2008). It may be benefitable to identify strengths and weaknesses prior to the implementation phase, as also recommended by others (Simpson and Dansereau 2007; Billsten et al. 2018).

## Conclusions

All participating schools in the X:IT II study possessed sufficient levels of general capacity. Participating schools with high overall readiness—defined as high motivation and high innovation-specific capacity—seemed to implement educational materials, parents’ meetings, smoke-free agreements, and smoking rules for teachers to a higher degree than schools which were low on both areas. There is still a long way to fully understand the complex issue of organizational readiness and implementation; however, this study adds to the literature by applying the heuristic to a school-based intervention and examining association to actual implementation.
